# Introduction to a mechanism for automated myocardium boundary detection with displacement encoding with stimulated echoes (DENSE)

**DOI:** 10.1259/bjr.20170841

**Published:** 2018-04-10

**Authors:** Julia Kar, Xiaodong Zhong, Michael V Cohen, Daniel Auger Cornejo, Angela Yates-Judice, Eduardo Rel, Maria S Figarola

**Affiliations:** 1 Departments of Mechanical Engineering and Pharmacology, University of South Alabama, Mobile, AL, USA; 2 MR R&D Collaborations, Siemens Healthcare Inc., Atlanta, GA, USA; 3 Department of Physiology, College of Medicine, University of South Alabama, Mobile, Al, USA; 4 Department of Biomedical Engineering, University of Virginia, Charlottesville, VA, USA; 5 Department of Radiology, University of South Alabama, 2451 USA Medical Center Drive, Mobile, AL, USA

## Abstract

**Objective:**

Displacement ENcoding with Stimulated Echoes (DENSE) is an MRI technique developed to encode phase related to myocardial tissue displacements, and the displacement information directly applied towards detecting left-ventricular (LV) myocardial motion during the cardiac cycle. The purpose of this study is to present a novel, three-dimensional (3D) DENSE displacement-based and magnitude image quantization-based, semi-automated detection technique for myocardial wall motion, whose boundaries are used for rapid and automated computation of 3D myocardial strain.

**Methods:**

The architecture of this boundary detection algorithm is primarily based on pixelwise spatiotemporal increments in LV tissue displacements during the cardiac cycle and further reinforced by radially searching for pixel-based image gradients in multithreshold quantized magnitude images. This spatiotemporal edge detection methodology was applied to all LV partitions and their subsequent timeframes that lead to full 3D LV reconstructions. It was followed by quantifications of 3D chamber dimensions and myocardial strains, whose rapid computation was the primary motivation behind developing this algorithm. A pre-existing two-dimensional (2D) semi-automated contouring technique was used in parallel to validate the accuracy of the algorithm and both methods tested on DENSE data acquired in (*N* = 14) healthy subjects. Chamber quantifications between methods were compared using paired *t*-tests and Bland–Altman analysis established regional strain agreements.

**Results:**

There were no significant differences in the results of chamber quantifications between the 3D semi-automated and existing 2D boundary detection techniques. This included comparisons of ejection fractions, which were 0.62 ± 0.04 *vs* 0.60 ± 0.06 (*p* = 0.23) for apical, 0.60 ± 0.04 *vs* 0.59 ± 0.05 (*p* = 0.76) for midventricular and 0.56 ± 0.04 *vs* 0.58 ± 0.05 (*p* = 0.07) for basal segments, that were quantified using the 3D semi-automated and 2D pre-existing methodologies, respectively. Bland–Altman agreement between regional strains generated biases of 0.01 ± 0.06, –0.01 ± 0.01 and 0.0 ± 0.06 for the radial, circumferential and longitudinal directions, respectively.

**Conclusion:**

A new, 3D semi-automated methodology for contouring the entire LV and rapidly generating chamber quantifications and regional strains is presented that was validated in relation to an existing 2D contouring technique.

**Advances in knowledge:**

This study introduced a scientific tool for rapid, semi-automated generation of clinical information regarding shape and function in the 3D LV.

## INTRODUCTION

The quantification of cardiac chamber dimensions and function is the foundation of cardiac imaging, of which MRI is the most reliable modality, providing superior resolution for soft-tissue contrast and multiplanar information that cannot be delivered by other modalities.^1–4^ Over the last few decades, specific MRI sequences have been developed for detecting cardiac cycle-based tissue motion, including spatial modulation of magnetization (SPAMM) or tissue-tagging, Displacement ENcoding with Stimulated Echoes (DENSE) and others.^5–10^ A particular matter of interest in relation to processing efficiency is automating the detection of myocardial wall motion for faster chamber quantification and functional assessment in the left-ventricle (LV). In this regard, DENSE is a sequence that can ideally be used to automate all computations, including boundary detection, determination of functional parameters as well as chamber quantifications, an approach which was demonstrated in a two-dimensional (2D) semi-automated motion estimation study by Spottiswoode et al.^7,9–11^

This study was conducted to investigate the feasibility of a highly automated, single-scan, MRI-based methodology for assessing full LV function (stain-based contraction and chamber quantifications), whose unprecedented rapid processing time (3.5 min approximately) might be of significant advantage in clinical applications. To achieve this goal, a novel and fast three-dimensional (3D) semi-automated methodology was used that primarily tracks LV boundary motion (during cardiac systole) using phase-encoded 3D displacement data recorded with the navigator-gated spiral DENSE MRI sequence.^11–15^ The displacement-based boundary search is further reinforced with a histogram distribution-based, multilevel thresholding-based image compression approach called Otsu’s method that was applied to identify myocardial tissue according to a discrete range of quantized indices.^16, 17^ In this regard, presented here is the first 3D, single-scan DENSE study that applies spatiotemporal displacements and the semi-automatedly detected LV boundaries toward rapid and automated 3D strain analysis and generation of surface strain maps.^9, 10^ Validation is provided by comparing the LV chamber quantifications and 3D strains computed with this new 3D methodology to those generated using the fully validated 2D boundary detection approach developed by Spottiswoode et al.^18–22^

## METHODS AND MATERIALS

### Human subject recruitments

#### DENSE acquisition and protocols

Navigator-gated 3D DENSE data were acquired with displacement encoding applied in two orthogonal in-plane directions and one through plane direction. A flexible, anterior 6-channel body matrix radiofrequency coil (Siemens Healthcare, Erlanger, Germany) and the table-mounted spine matrix radiofrequency coil were used for receiving signals.^11, 12,19^ Typical imaging parameters included field of view of 380 × 380 mm^2^, echo time of 1.04 ms, repetition time of 15 ms, matrix size of 128 × 128 × 19, 2.97 × 2.97 × 5 mm^3^ voxel size, 21 cardiac phases, encoding frequency of 0.06 cycles mm^-1^, simple 4-point encoding and 3-point phase cycling for artifact suppression.^14, 15,23^ Given that three heartbeats are needed to acquire a complete set of spirals for a single displacement encoding direction and a single phase cycling point, the number of navigator-accepted heartbeats to complete a single partition in 3D is (4 × 3 × 3) = 36 heartbeats.^15^ Hence, the acquisition time per subject was about 15 min depending on the heart rate and navigator acceptance rate of individual subject. Continuous monitoring of heart rates and blood pressures were conducted during the scans for all subjects.

### Image quantization

**Figure 1.**
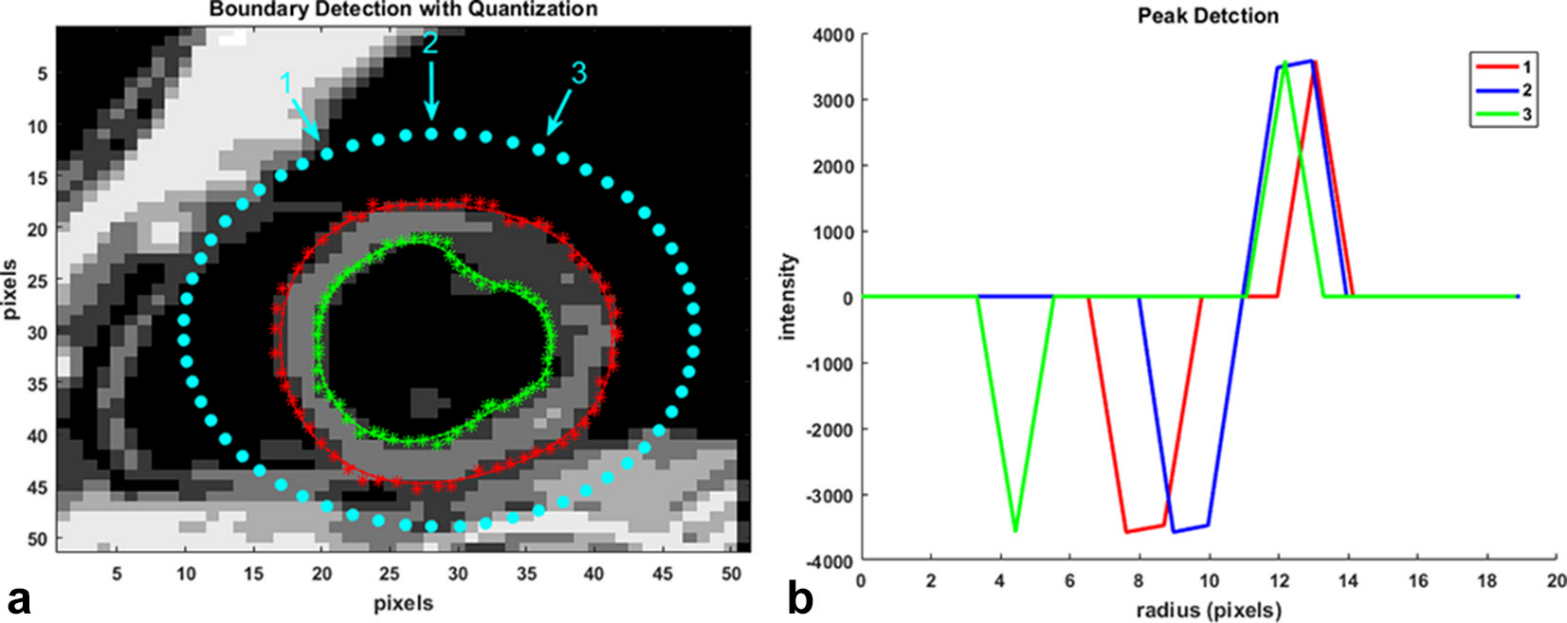
(a) Boundary detection with the 3D semi-automated technique starts with a bounding ellipse (outer dotted ellipse) that locates points on the myocardial boundaries based on pixelwise intensity gradients in an inwards, radial direction. Detection starts with the quantized reference image at end-diastole and is continued based on phase-unwrapped displacement information in subsequent timeframes. Dotted red line is the semi-automated epicardial contour before applying the LOESS curve fitting technique and starred red line is the one after the LOESS fit. Dotted green line is the pre-LOESS fit semi-automated endocardial contour and starred green line is the one after the LOESS fit. (b) Examples of pixel intensity-based gradients in the radial direction whose peaks are used to locate epicardial and endocardial boundary points. 3D, three-dimensional.

**Figure 2.**
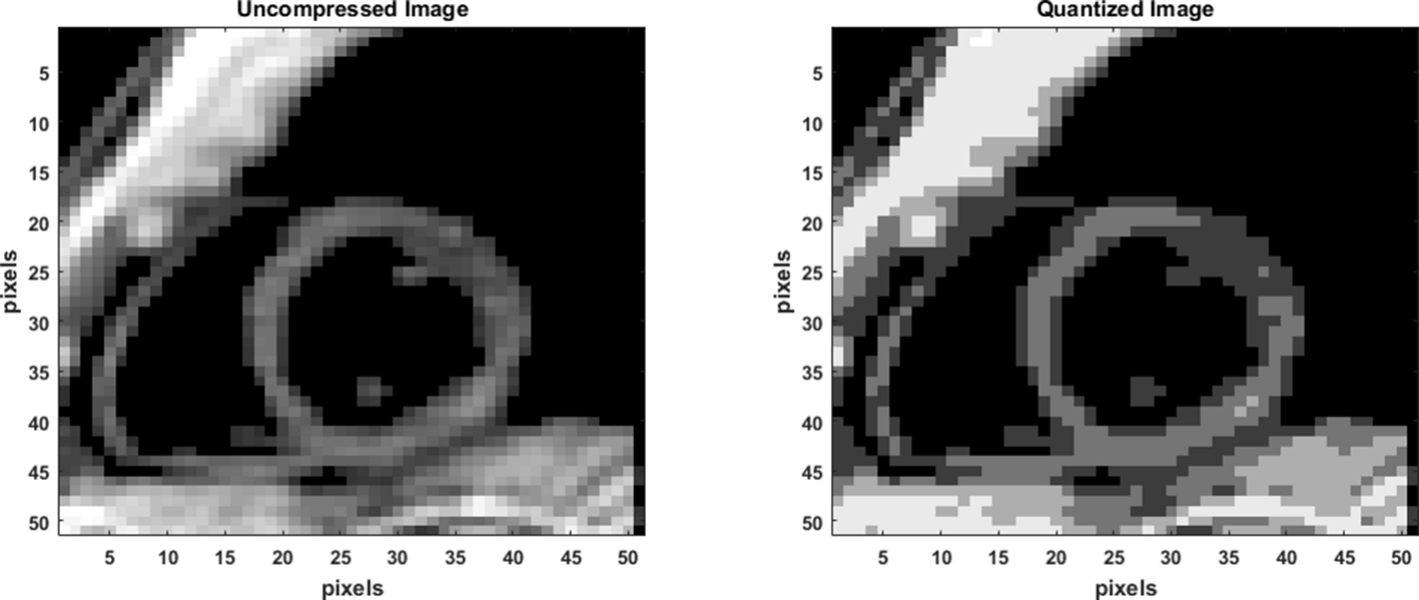
Quantization with a Lossy irreversible compression process. The original image shown has been quantized to seven classes of pixels and six thresholds using the non-uniform, histogram-based Otsu’s method.

Identification of the LV boundary contours in each short axis slice (image) at a reference timeframe (end-diastole) is the first step in this semi-automated process. This process occurred by the operator selecting an ellipsoidal region of interest (ROI) in the most basal short-axis slice (shown in Figure 1) at end-diastole, and followed by propagating that ROI for all other slice positions between base and apex. The LV boundaries and intramural tissue are then identified using an image quantization process, where a threshold image is formed with a distinct profile of the short-axis LV (Figure 2). Prior to quantization, a basic noise removal scheme was applied and consisted of a Gaussian filter (convolution matrix) of kernel size 3 × 3. Quantization is a commonly used image processing tool, a lossy (or irreversible) compression technique that compresses a range of colors (or gray values) to a single quantum value. A non-uniform quantization scheme based on image histogram, called Otsu’s Method, was used such that the resulting image comprised of pixels from the most commonly occurring intensities.^16, 17^ In its most basic form, the Otsu algorithm separates two classes of pixels based on a bimodal histogram (foreground and background), and calculates the optimum threshold separating the two classes such that their interclass variance is maximal.^16, 17^ The maximizing interclass variance, *σ*_*b*_, in Otsu’s method, in an image of pixel intensities ranging from *i* = 1, .., L, is given by,

$$\sigma_{b}^{2}=\omega_{0}\left(t\right)\omega_{1}\left(t\right){\left(\mu_{0}-\mu_{1}\right)}^{2}$$

where weights *ω*_0_ and *ω*_1_ are the probabilities of the two classes separated by a threshold, *t*, and defined as

$$\omega_{0}\left(t\right)=\sum_{i=1}^{t}p_{i}\;\omega_{1}\left(t\right)=\sum_{i=t+1}^{L}p_{i}$$

where *p*_i_ is the probability of intensity *i* in the image and *µ*_0_ and *µ*_1_ are the class means given by,

$$\mu_{0}=\sum_{i=1}^{t}i\frac{p_{i}}{\omega_{0}\left(t\right)}\;\;\;\;\;\;\mu_{1}=\sum_{i=t+1}^{L}i\frac{p_{i}}{\omega_{1}\left(t\right)}$$

where *L* is the total number of bins in the original histogram.

The previous formula can be easily extended to multilevel thresholding as was done for this study. The multilevel method assumes that there are M-1 thresholds, (*t*_1_*, t*_2_, …, *t*_*M-*1_), which divide the original image into M classes.^16^ In this case, the maximizing interclass variance is given by,

$$\sigma_{b}^{2}=\sum_{k=1}^{M}\omega_{k}\;{\left(\mu_{k}-\mu_{T}\right)}^{2}$$

$$\;\;\mu_{k}=\sum_{i\in C_{k}}^{}i\frac{p_{i}}{\omega \left(k\right)}$$

$$\omega_{k}=\sum_{i\in C_{k}}^{}p_{i}$$

where *ω*_k_ is regarded as the zero-order cumulative moment of the *k*th class *C*_k_, and *µ*_T_ is the cumulative mean for the entire image.

Example of quantization with multilevel thresholding of a short-axis slice based on histogram data is shown in Figure 2. The effectiveness of the thresholding scheme (number of classes selected) for each image in the entire stack can be computed by iterating through a range for the number of thresholds and the computation of an effectiveness metric. The formula for the effectiveness metric, which is a value in the range 0.0–1.0, is given by,

$$EM=\;\frac{\sigma_{b,max}^{2}}{\sum_{i=1}^{L}{\left(ip_{i}-\;\mu_{T}\right)}^{2}}$$

The lower bound in Equation (7) is attainable only by images having a single gray level, and the upper bound is attainable only in binary images.

### Image gradients from thresholding

One of the fundamental mechanisms of edge detection in images involve identifying sudden changes in intensities in a cluster of local pixels. This concept can be applied towards detecting myocardial boundaries in a quantized image with a limited number of pixel intensities. When pixel-based intensity gradients are computed in a radial direction, starting with points on a bounding ellipse and moving across the LV myocardium, local peaks are created where there are abrupt changes in intensities, as shown in Figure 1. This study used these peaks to form the LV anatomical boundaries along with a shape constraint implemented by minimizing an error function. The shape constraint for forming the myocardial boundary consisted of fitting a non-parametric regression LOcal regrESSion (LOESS) curve to the gradient peaks located by the algorithm and computing the sum of squares of the differences between the peaks and their corresponding LOESS points.^24, 25^ To briefly explain the LOESS fit, for each boundary point a low-degree polynomial is least square fitted to a subset of boundary points, with explanatory variable values near the point whose response is being estimated.^24, 25^ In addition, the subsets of points used for each weighted least squares fit are determined by a nearest neighbors algorithm and a smoothing parameter, α (typical range used was 0.25–0.3), which determined how much of the data points would be used to fit each local polynomial. In this way, and by specifying a maximum number of iterations, a new peak was selected every time it reduced the following error term, ε, defined as,

$$\varepsilon =\sum_{n=1}^{N}{\left(r_{p}\left(x,y\right)-r_{L}\left(x,y\right)\right)}^{2}$$

where *r*_p_ is the location of the 2D slice-based peak and *r*_L_ is the location of the LOESS point.

### Boundary detection with displacement encoding

**Figure 3.**
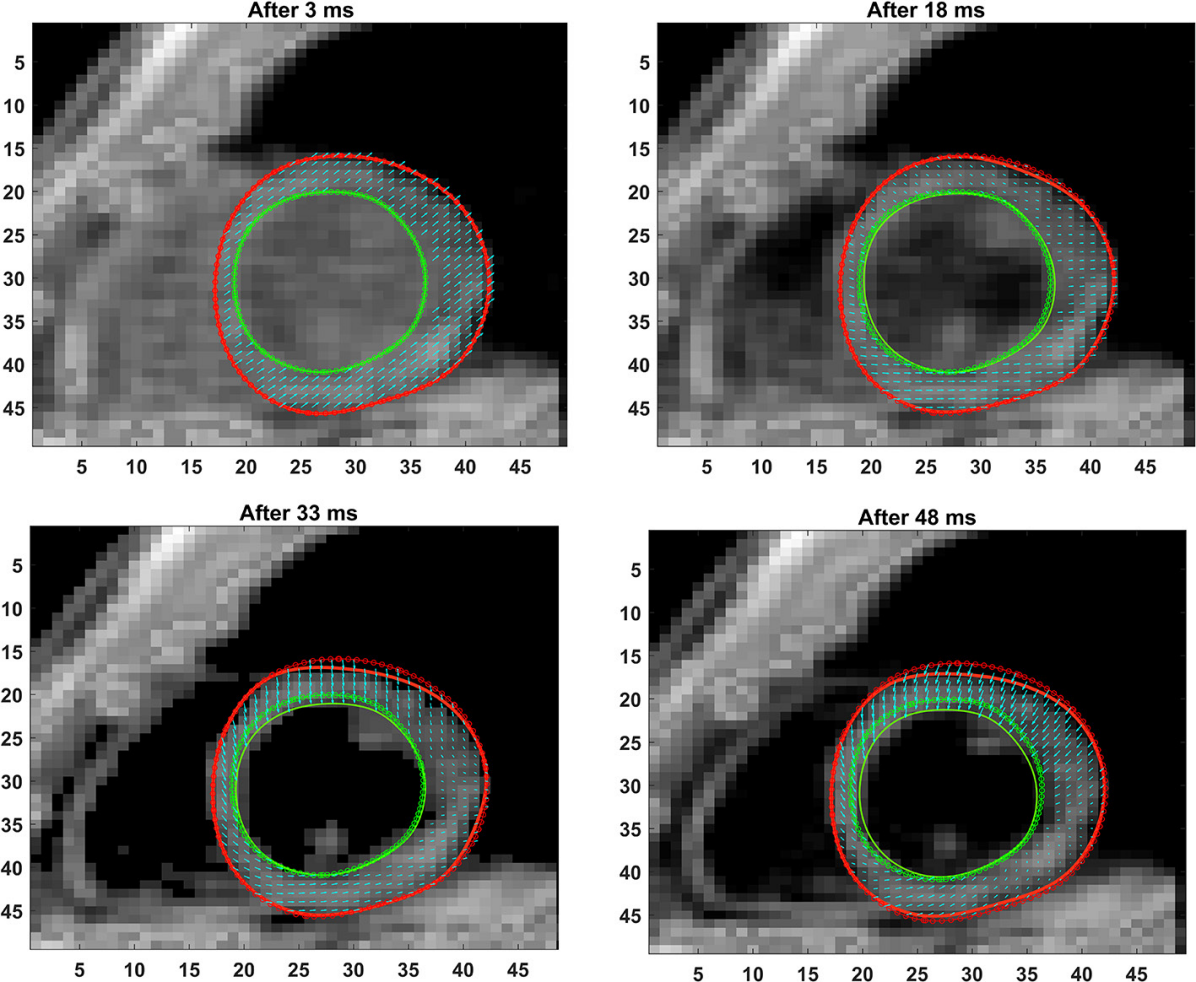
Shown are the progressive phase-unwrapping based pixelwise displacements within the myocardium boundaries from end-diastole (dotted lines) through subsequent timeframes (solid lines) untill 48 ms in time. The mean incremental displacements in neighboring pixels (between consecutive timeframes) were applied to determine the direction of motion at each pixel for a new timeframe and the location of the new boundary points determined.

**Figure 4.**
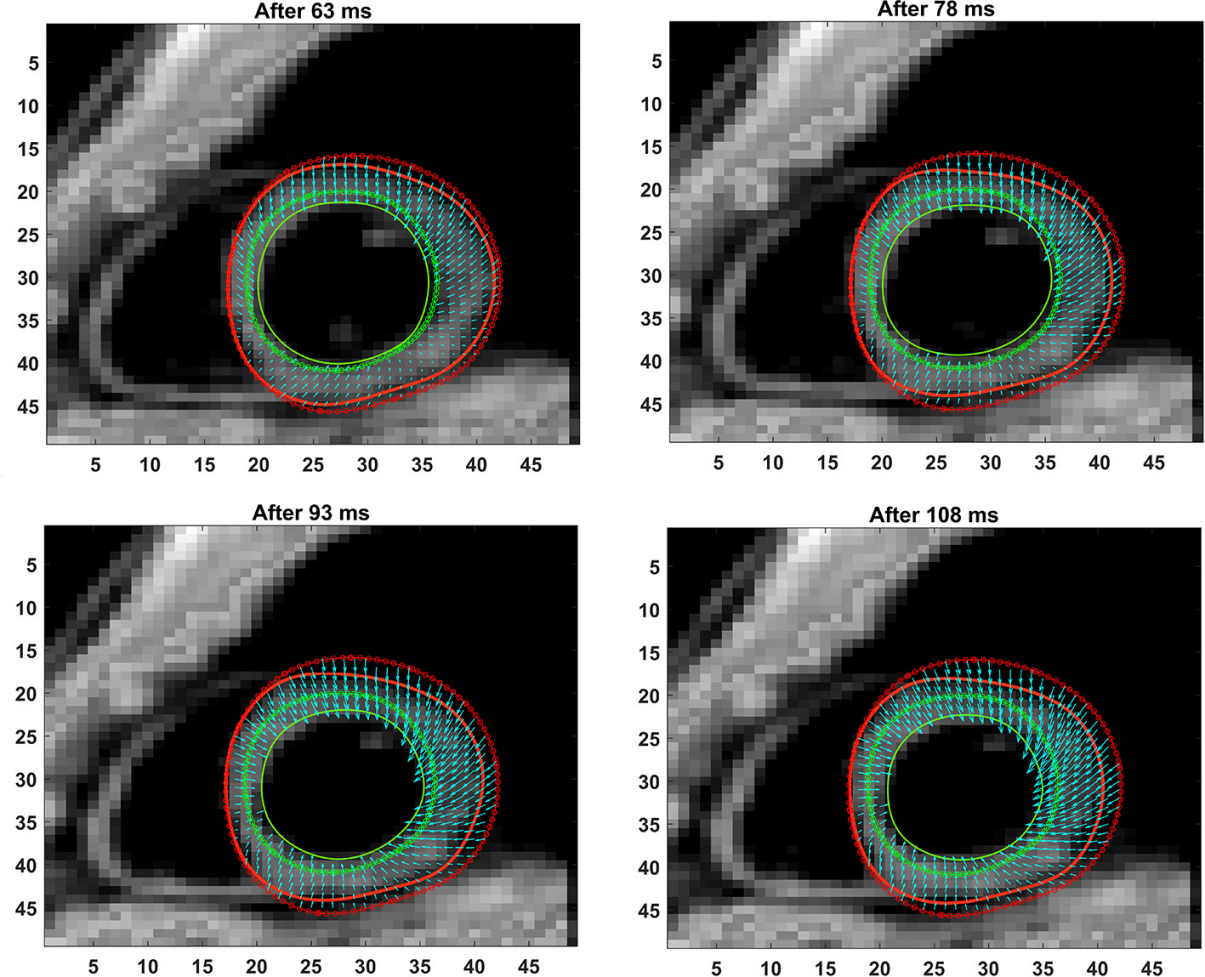
Shown are the progressive phase-unwrapping based pixelwise displacements within the myocardium boundaries for timeframes between 63 ms and 108 ms, following those in Figure 3.

**Figure 5.**
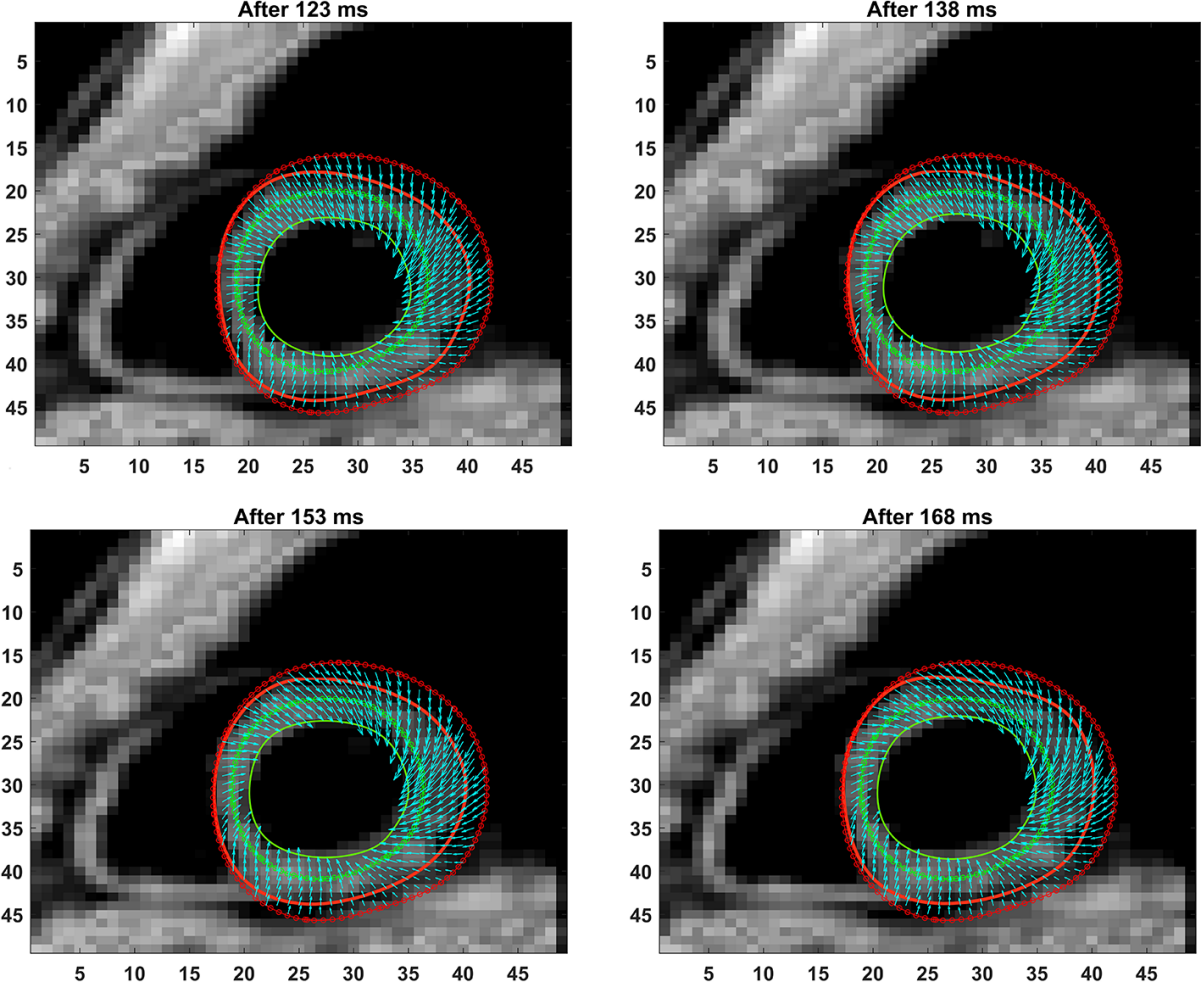
Shown are the progressive phase-unwrapping based pixelwise displacements within the myocardium boundaries for timeframes between 123 ms and 168 ms (end-systole), following those in Figure 4.

In this study, soft tissue deformation quantified by its influence on both signal amplitude and phase was the primary driver for assessing cardiac kinematics and boundary motion in a semi-automated manner. Precisely, the background to measuring deformation fields in DENSE is a well-established magnetic field gradients/Fourier Transform methods phenomenon called the pulsed gradient stimulated echo, incorporating motion encoding by employing a pair of pulsed field gradients.^26^ DENSE employs the pulsed gradient stimulated echo technique to encode tissue displacement in the phase of the stimulated echo, thus enabling the acquisition of pixelwise tissue displacements from which the semi-automated frame-by-frame myocardial boundary motion is finally derived. Hence, the new position of a boundary control point can be found by tracking pixels with displacements, whose tails map back to an original boundary control point at end-diastole.^11, 12,15^ Detailed descriptions for phase-to-displacement conversions with pixelwise phase unwrapping can be found in several previous publications, including our studies in DENSE-based strain validation.^10–12,15,18^ A final step involves refining the raw displacement data using temporal fitting of each pixel’s trajectory with Fourier (fifth order) basis functions as described in previous studies.^12, 15,18,19^ The series of images in Figures 3–5 show the incremental motion that occur at the pixel level and the morphology of the myocardial boundaries with LV contraction as the boundary points relocate to new positions. Ideally, the detection of a peak along a radial search path of threshold-based gradients, which has a displacement with its tail at an original (diastole) boundary point, provides the location of a boundary point in a new timeframe. In this regard, the search for a peak is limited to ±2 pixels in the radial path as a means of imposing frame-to-frame physiological limits. When a gradient peak is not detected in the radial path, a boundary point can still move if a pixel’s displacement tails back to an original boundary point, other boundary points in its neighborhood have moved and its quantization index is in the myocardial range. Alternatively, if a gradient peak is detected whose tail cannot be traced back to an original end-diastole boundary point, it can still be considered a point of boundary relocation if its tail can be traced back to within ±1 pixel of an original boundary point in end-diastole. The underlying assumption for the above is the superposition of a close-to-boundary material point on an original boundary point following contraction. In this way, the mean of the frame-to-frame increment in the displacement of a boundary point’s neighboring pixels can be approximated, and in conjunction with the new frame’s quantized image applied towards determining the material underlying a boundary point in the new timeframe. If the quantization index for a new boundary point location indicates non-myocardial material (*e.g.* blood pool or chest cavity), the point is relocated to the closest pixel whose quantization index falls within the myocardial range. LOESS curve fits are once again applied to each boundary following the finding of new material-based boundary points in each subsequent timeframe.

Following the generation of boundaries for all partitions and time frames, as well as 3D reconstruction using existing techniques, measurements for chamber quantifications for specific parameters, such as LV wall thickness, end-diastolic diameter (EDD), end-systolic diameter (ESD), end-diastolic volume (EDV), end-systolic volume (ESV), ejection fraction (EF) and LV mass were conducted using guidelines provided in gold-standard MRI studies.^4,27–29^ Individuals who have 2 or more years of experience in DENSE post-processing randomly performed boundary detections with this 3D semi-automated process. The individuals consisted of two graduate students (one each from engineering and medicine) and an individual with a doctorate in biomedical engineering. Hence, an individual who has a graduate background in science or technology is suitably qualified to perform this post-processing.

### The 2D Spottiswoode et al semi-automated technique

Given here is a summarized description of the 2D automated DENSE-based boundary detection technique by Spottiswoode et al in reference to which the currently proposed 3D semi-automated technique was validated.^10, 12^ A more comprehensive summary regarding the Spottiswoode et al approach to automatic spatiotemporal tracking of 2D myocardial material points from cine DENSE displacement observations can be found in their original articles. With this technique, epicardial and endocardial contours must be initialized manually for each partition prior to computing phase-based displacements for detecting boundaries in subsequent time frames. The displacement vector starting points in subsequent phases is, therefore, determined by this myocardium at *t*_0_, and tracking these points though time identified the position of the myocardium in the following frames. However, they found that obtaining suitable motion trajectories from noisy displacement fields required a number of refinements to the motion tracking algorithm. Hence, to accurately track myocardial tissue motion, noisy displacement vectors were first removed using a modulus deformation mask, which was obtained for each frame by combining orthogonal spatial derivatives of the displacement fields.^10^ Remaining noisy vectors were then eliminated based on their deviation from the mean magnitude and angle of nearby vectors and a cluster of vectors with tails nearest to each starting point at *t*_0_ selected to refine displacement.^10, 12^ Trajectories were further improved by applying temporal fitting as described in Spottiswoode et al with periodic Fourier basis functions fitted to each horizontal and vertical components of motion as a function of time.^10, 12^ Reconstructing the 3D LV geometry with the estimated 2D myocardial boundaries was conducted using the same technique as used for this study’s 3D semi-automated process.^19^ Following this, pixelwise 3D strain tensors were computed using the meshfree Radial Point Interpolation Method (RPIM) methodology and the strains averaged for the 16 standard myocardial segments. The generation of myocardial boundaries using this technique was performed in a random fashion by the same individuals who conducted the boundary assessment for the new 3D semi-automated technique. A summarized comparison between this study’s 3D semi-automated technique and the Spottiswoode et al methodology is given in Table 1.

**Table 1.** A comparison of techniques between the existing 2D Spottiswoode et al and the new 3D semi-automated technique for motion-based LV wall detection

**Attribute**	**2D Spottiswoode et al method**	**3D semi-automated method**
Modality/sequence	MRI/DENSE	MRI/DENSE
Dimension of processed data	2D	3D
Primary contour detection algorithm	Phase-encoded displacements	Phase-encoded displacements
Secondary contour detection algorithm	Modulus deformation mask	Multi-threshold based quantization
Chamber quantifications	No direct method	EDV, ESV, EF
Initial ROI	Bounding torus	Bounding ellipsoid
Processing speed	1–2 min per 2D slice	<5 min for entire LV
Manual modification	Possible	Possible
Strain estimations	Slice-based	Entire volume
Surface strain maps	None	Possible

2D, two-dimensional; 3D, three-dimensional; DENSE, Displacement Encoding with Stimulated Echoes; EDV, end-diastolic volume; EF, ejection fraction; ESV, end-systolic volume; LV, left-ventricle; ROI, region of interest.

### Meshfree strain analysis

Three-dimensional strain parameters (radial, circumferential and longitudinal) were computed using RPIM at each voxel in patient-specific MRI-based reconstructed 3D grid geometries. RPIM is a numerical analysis technique based on the Galerkin weak form that uses radial basis functions as 3D meshfree shape functions and facilitates fast multidimensional computation of Lagrangian strains.^18–20,22,30,31^ Since RPIM shape functions have the Kronecker delta functions property, essential boundary conditions are simply enforced, as otherwise done in finite element analysis. Hence, combining the advantages of DENSE with RPIM provides fast and effective 4D spatiotemporal analysis of strain, readily computed at a given voxel, and without the tedium of keeping track of tagged data or remeshing interventions that otherwise prolong modelling time in traditional finite element analysis.^12, 14^ Extensive descriptions of both RPIM and its use in computing 3D LV strains is outlined in previous literature, which in particular details the use of the Multiquadrics as radial basis function shape function that ensure C^1^ continuity.^18–22,31^ For the final comparison of strains between the two methodologies, the LV was segmented into 16 standard regions and the mean strain estimated for each from the grid-point values.

### Validation and statistical analysis

The LV chamber quantification results from the 3D semi-automated and Spottiswoode et al contouring techniques were compared using paired *t*-tests, where the key parameters included wall thickness, EDD, ESD, EDV, ESV, EF and LV mass. The estimated parameters were also compared to gold-standard measurements reported in previous MRI studies.^4,32–34^ Additionally, Bland–Altman analysis provided the measure of agreement between RPIM-based 3D strains, in the radial, circumferential and longitudinal directions, from the two contouring techniques. The strain results were also non-empirically compared to measurements reported in previous tissue tagging studies and to those from validation studies in 3D DENSE.^18, 19,35^

## RESULTS

### Subject details

The average age of the subjects was 30.5 ± 7.8 years and body weight was 65.8 ± 9.6 kgs . Monitored mean heart rate from all studies was 66.6 ± 8.0 bpm while mean blood pressure was 120.0 ± 16.7/77.3 ± 15.1 mmHg.

### Experimental and statistical results

The full LV chamber quantification, from both 3D semi-automated and Spottiswoode et al boundary contouring methods, are given in Table 2. Among measurements of fundamental parameters, comparable values in thickness were seen between 3D semi-automated and Spottiswoode et al boundary methods in the apical segment at 7.6 ± 1.1 *vs* 7.9 ± 1.5 mm (*p* = 0.12), midventricular segment at 7.7 ± 1.4 *vs* 7.8 ± 1.3 mm (*p* = 0.69) and basal segment at 7.8 ± 1.6 *vs* 7.6 ± 1.5 mm (*p* = 0.83), respectively. Other fundamental measurements for which significant differences were not found between the methodologies include apical EDD (*p* = 0.19), midventricular EDD (*p* = 0.63), basal EDD (*p* = 0.10), apical ESD (*p* = 0.14) and midventricular ESD (*p* = 0.52), with the only exception seen in the basal ESD (*p* = 0.04). Next, the fundamental dimensions were applied towards computing 3D chamber dimensions such as volume, ejection fraction and mass for both techniques, estimates of which are given in Table 2. Figures 3–5 show contour maps of LV myocardial boundaries estimated with the 3D semi-automated boundary detection algorithm after each acquisition interval (*t* = 15 ms). Also shown in Figures 3–5 and as well as provided as a movie in the supplementary material are the directions of pixel-based increments in deformation after each interval with reference points at end-diastole. A movie of the changing boundary contours related to Figures 3–5 is also provided.

**Table 2.** Paired *t*-test comparisons of LV chamber parameters (given as mean ± SD) estimated with the new 3D semi-automated and existing 2D Spottiswoode et al contouring techniques in *N* = 14 normal subjects

Parameter	3D semi-automated method (mean ± SD)	2D Spottiswoode et al method (mean ± SD)	*p*-value
Apical thickness (mm)	7.6 ± 1.1	7.9 ± 1.5	0.12
Midventricular thickness (mm)	7.7 ± 1.4	7.8 ± 1.3	0.69
Basal thickness (mm)	7.8 ± 1.6	7.6 ± 1.5	0.83
Apical EDD (cm)	3.2 ± 0.3	3.5 ± 0.6	0.19
Midventricular EDD (cm)	3.9 ± 0.4	4.0 ± 0.5	0.63
Basal EDD (cm)	4.7 ± 0.6	4.5 ± 0.7	0.10
Apical ESD (cm)	2.2 ± 0.3	2.4 ± 0.6	0.14
Midventricular ESD (cm)	2.7 ± 0.4	2.8 ± 0.5	0.52
Basal ESD (cm)	3.6 ± 0.6	3.3 ± 0.7	0.04^*a*^
Apical EDV (cm^3^)	21.8 ± 4.6	26.2 ± 8.5	0.11
Midventricular EDV (cm^3^)	32.3 ± 6.5	33.9 ± 8.6	0.56
Basal EDV (cm^3^)	49.6 ± 12.3	44.0 ± 12.7	0.13
Apical ESV (cm^3^)	8.4 ± 2.6	11.0 ± 4.9	0.09
Midventricular ESV (cm^3^)	13.2 ± 3.7	14.2 ± 4.9	0.53
Basal ESV (cm^3^)	22.3 ± 7.2	19.1 ± 7.2	0.14
Apical EF	0.62 ± 0.04	0.60 ± 0.06	0.23
Midventricular EF	0.60 ± 0.04	0.59 ± 0.05	0.76
Basal EF	0.56 ± 0.04	0.58 ± 0.05	0.07
Apical mass (g)	38.3 ± 5.8	45.2 ± 12.5	0.08
Midventricular mass (g)	53.1 ± 10.9	55.3 ± 11.8	0.29
Basal mass (g)	74.2 ± 15.5	66.6 ± 15.8	0.10

2D, two-dimensional; 3D, three-dimensional; EDD, end-diastolic diameter; EDV, end-diastolic volume; EF, ejection fraction; ESD, end-systolic diameter; ESV, end-systolic volume; SD, standard deviation.

a Indicates the only significant difference between methodologies.

**Figure 6.**
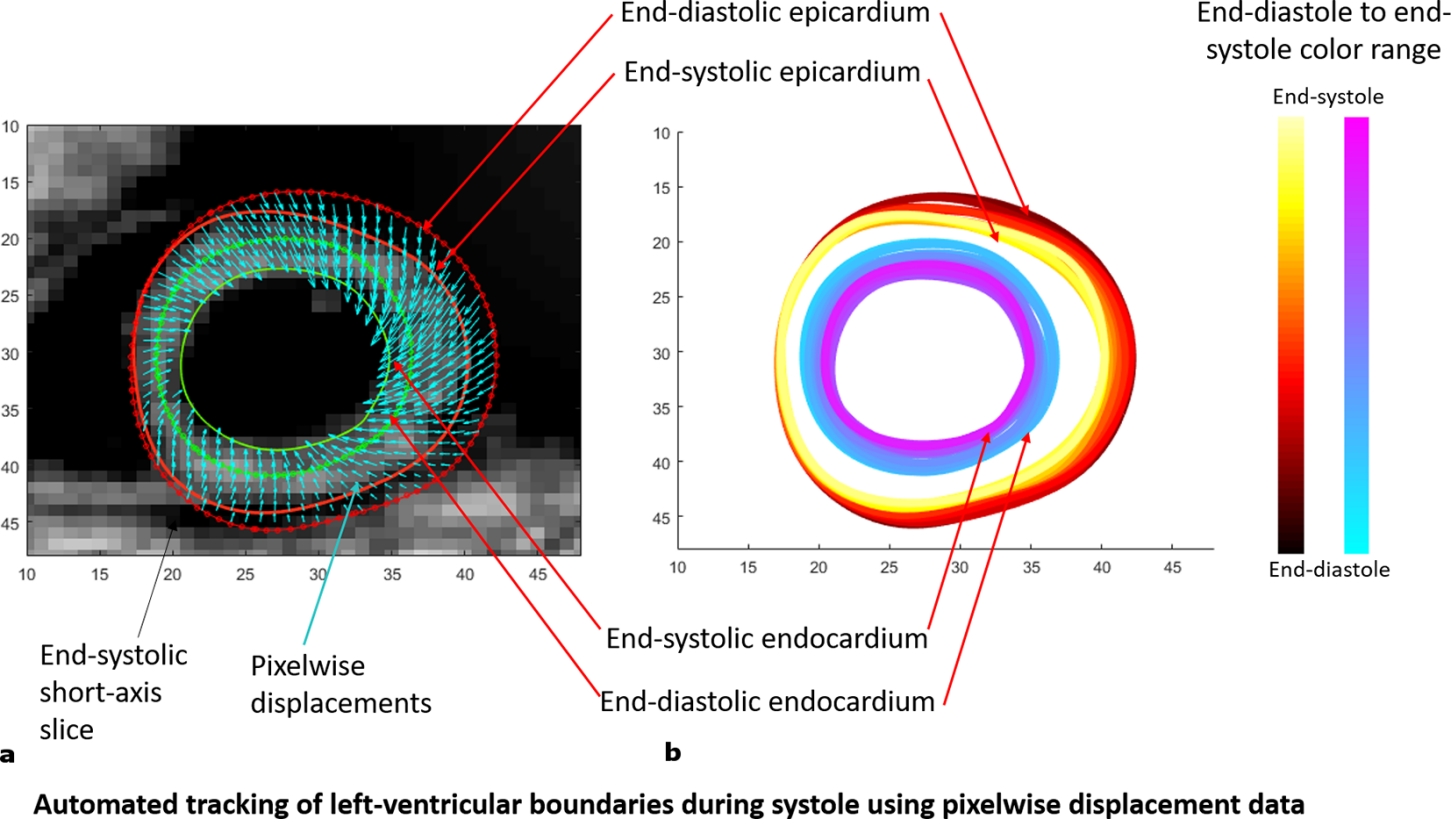
(a) The end-systolic boundaries (solid red and green lines) shown in comparison to the end-diastolic boundaries (dotted red and green lines). Finding a control point’s motion at each timeframe occur by tracking pixels with displacements (shown in cyan), whose tails map back to an original boundary control point at end-diastole. (b) Morphology of the two myocardial boundaries between end-diastole and end-systole using the 3D semi-automated methodology.

**Figure 7.**
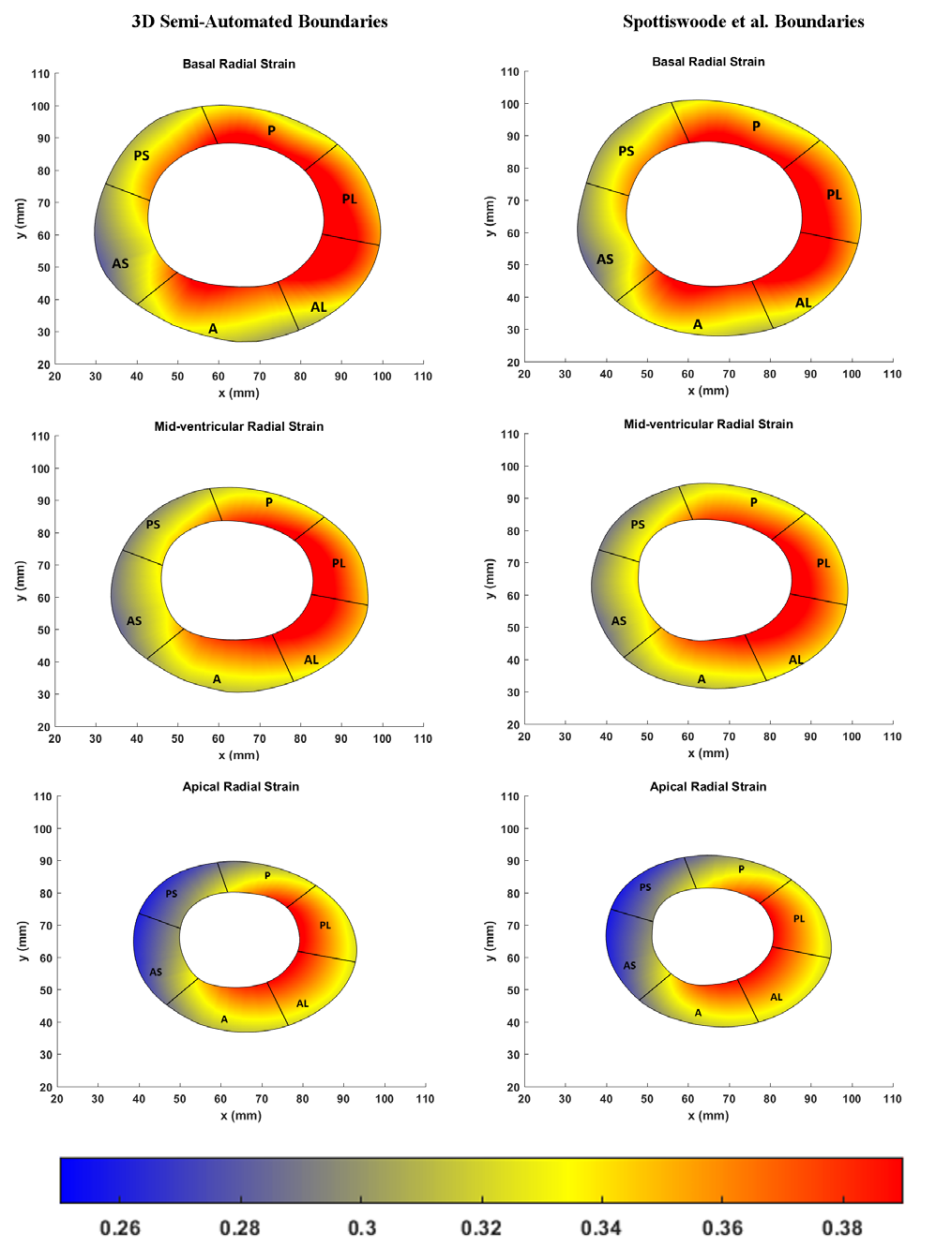
Comparison of basal (top), midventricular (middle) and apical (bottom) boundary contours with surface maps of radial strains between the new 3D semi-automated method (left) and the Spottiswoode et al. (right) method. Abbreviations: 3D, three-dimensional; A, anterior; AS, anteroseptal; PS, posteroseptal; P, posterior; PL, posterolateral; AL, anterolateral.

**Figure 8.**
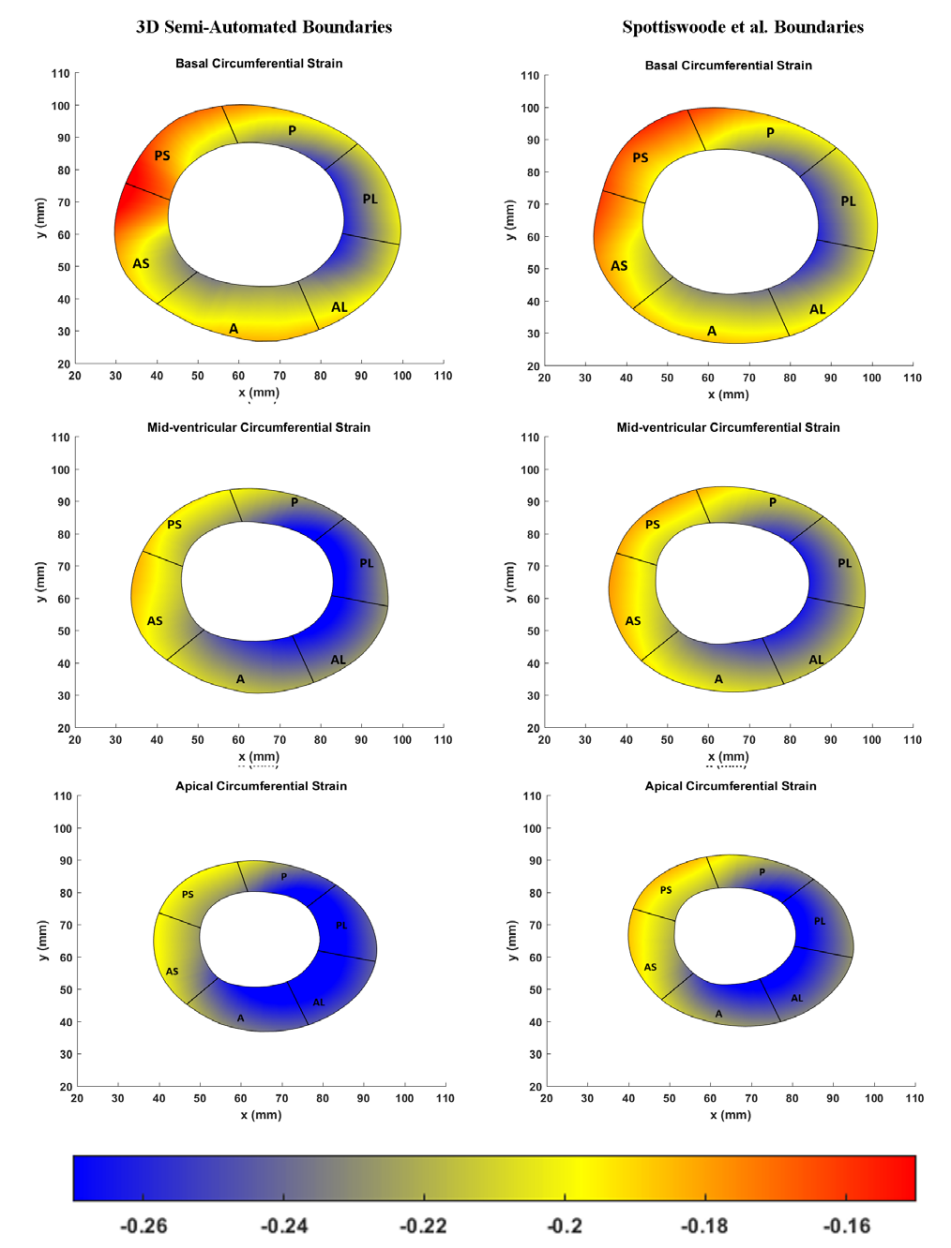
Comparison of basal (top), midventricular (middle) and apical (bottom) boundary contours with surface maps of circumferntial strains between the new 3D semi-automated method (left) and the Spottiswoode et al. (right) method. Abbreviations: 3D, three-dimensional; A, anterior; AS, anteroseptal; PS, posteroseptal; P, posterior; PL, posterolateral; AL, anterolateral.

**Figure 9.**
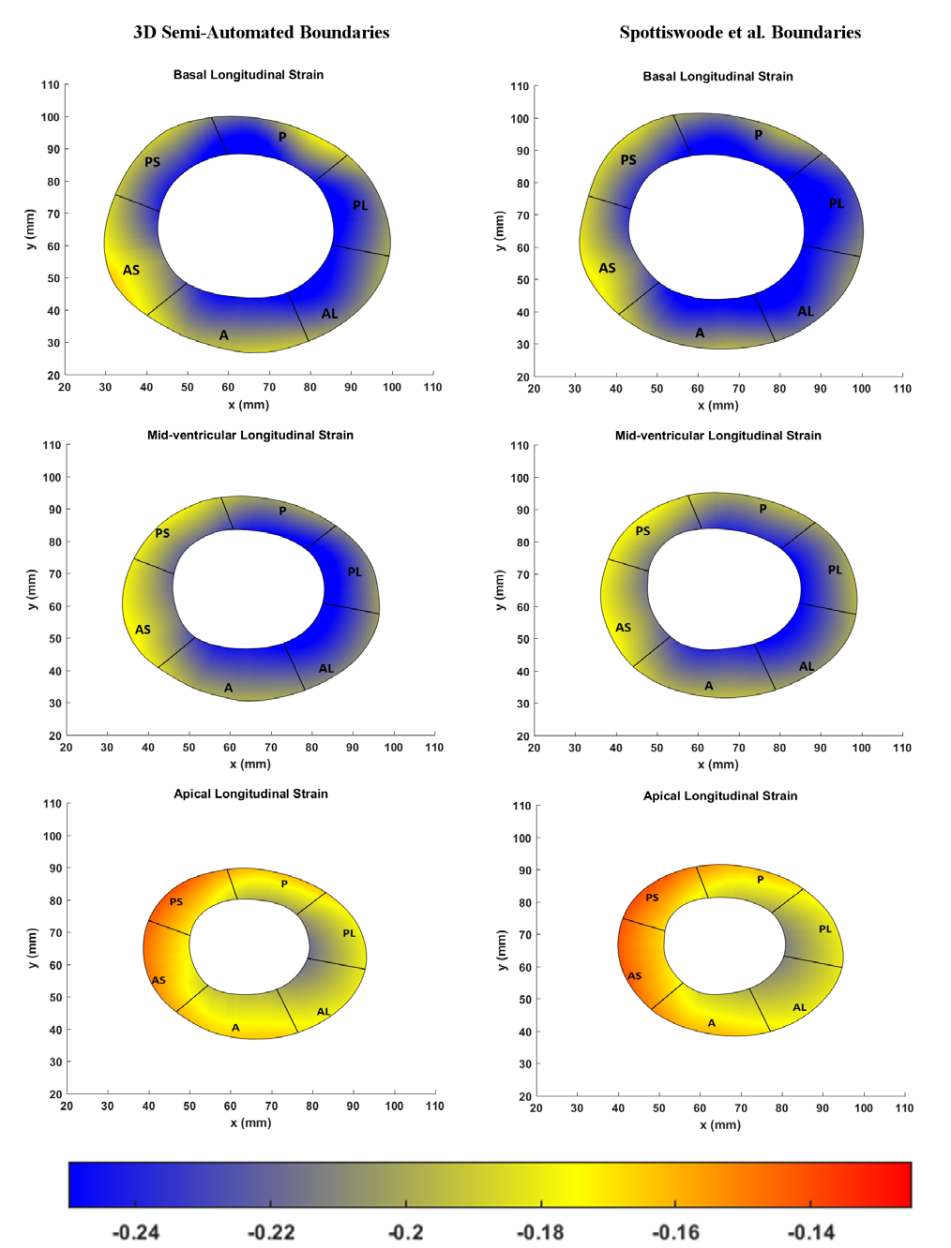
Comparison of basal (top), midventricular (middle) and apical (bottom) boundary contours with surface maps of longitudinal strains between the new 3D semi-automated method (left) and the Spottiswoode et al. (right) method. Abbreviations: 3D, three-dimensional; A, anterior; AS, anteroseptal; PS, posteroseptal; P, posterior; PL, posterolateral; AL, anterolateral.

The left hand side of Figure 6 shows the boundary location at end-systole relative to the position of the boundary at end-diastole while boundary morphology, at time intervals of 15 ms, is shown on the right hand side of Figure 6. Bland–Altman agreements between regional strain magnitudes computed with the 3D semi-automated and Spottiswoode et al boundaries in terms of biases ± limits of agreement were 0.01 ± 0.06, –0.01 ± 0.01 and 0.0 ± 0.06 for the radial, circumferential and longitudinal directions, respectively. Figures 7–9 are a comparison of the boundaries generated with the contouring techniques in typical basal, midventricular and apical slices and shows the cross sectional maps of radial (Figure 7), circumferential (Figure 8) and longitudinal (Figure 9) strains.

**Figure 10.**
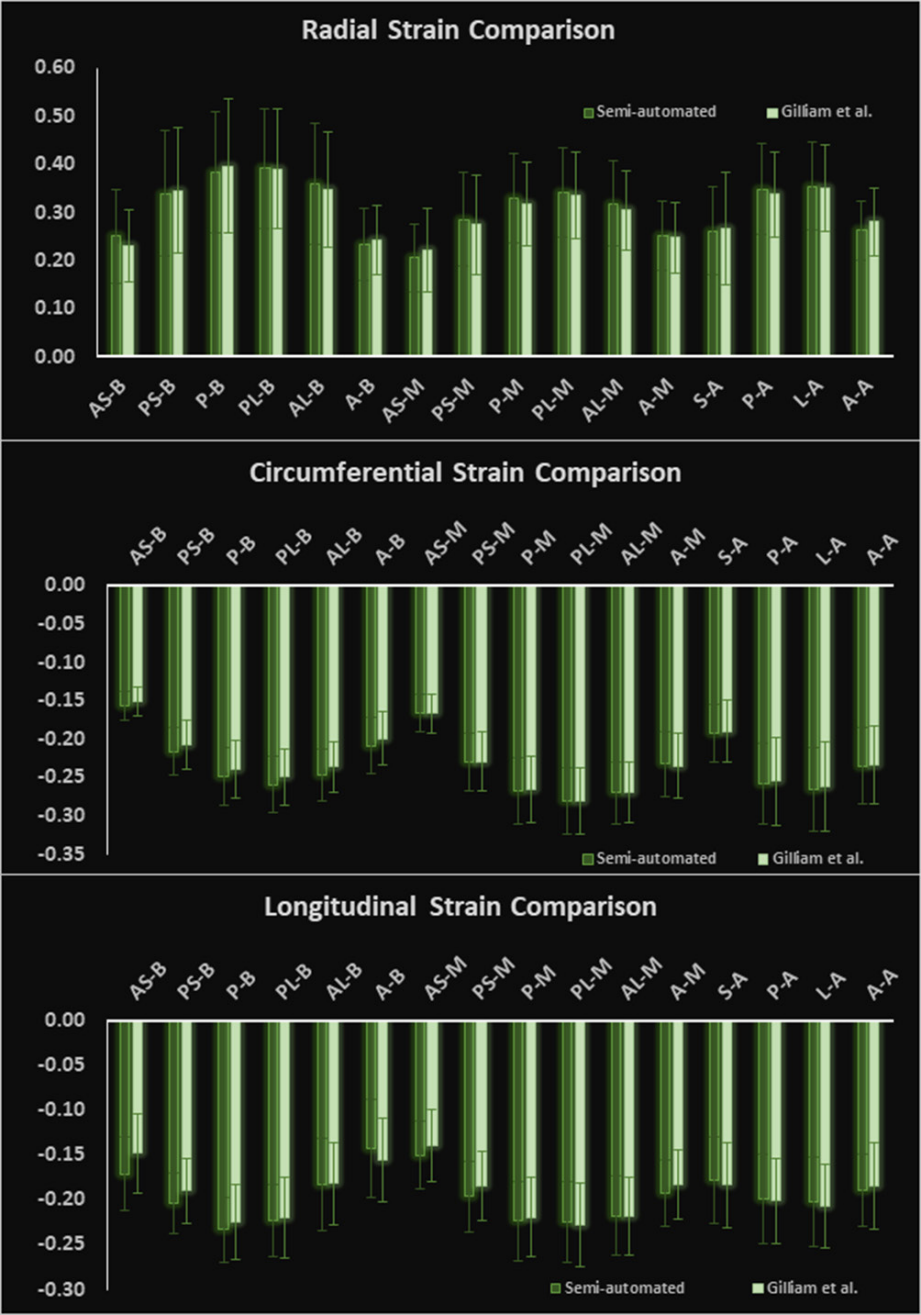
Comparison of 3D regionally averaged (a) circumferential, (b) radial and (c) longitudinal strains between the new 3D semi-automated and 2D Spottiswoode et al contouring techniques in *N* = 14 normal subjects. Strain was computed with the RPIM applied to 3D LV geometries and phase-unwrapped displacements. Abbreviations: 2D, two-dimensional; 3D, three-dimensional; A, anterior; A, apical; AL, anterolateral; AS, anteroseptal; B, basal; MV, midventricular; P, posterior; PL, posterolateral; PS, posteroseptal; RPIM, Radial Point Interpolation Method.

**Figure 11.**
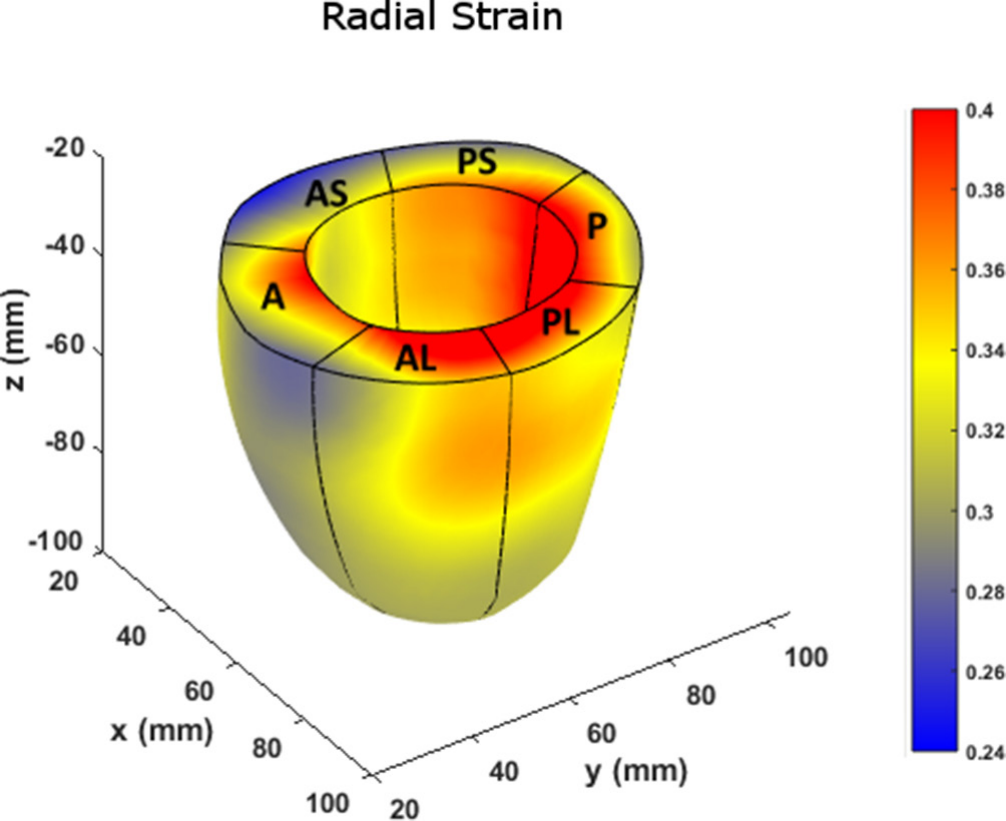
Surface map of radial strain computed with RPIM on LV geometries, reconstructed from boundaries detected with the newly introduced 3D semi-automated myocardial contouring technique. Abbreviations: 3D, three-dimensional; A, anterior; A, apical; AL, anterolateral; AS, anteroseptal; B, basal; LV, left-ventricular; MV, midventricular; P, posterior; PL, posterolateral; PS, posteroseptal; RPIM, Radial Point Interpolation Method.

**Figure 12.**
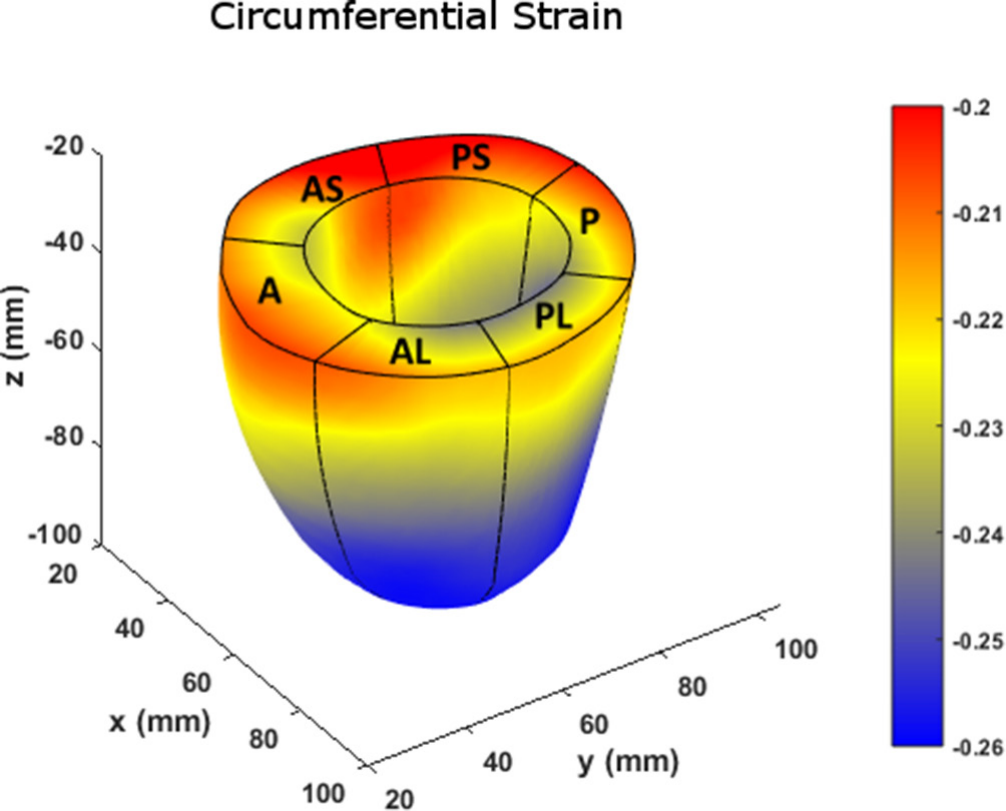
Surface map of circumferential strain computed with the RPIM method on LV geometries, reconstructed from boundaries detected with the newly introduced 3D semi-automated myocardial contouring technique. Abbreviations: 3D, three-dimensional; A, anterior; AS, anteroseptal; PS, posteroseptal; P, posterior; PL, posterolateral; AL, anterolateral; LV, left-ventricular; RPIM, Radial Point Interpolation Method.

**Figure 13.**
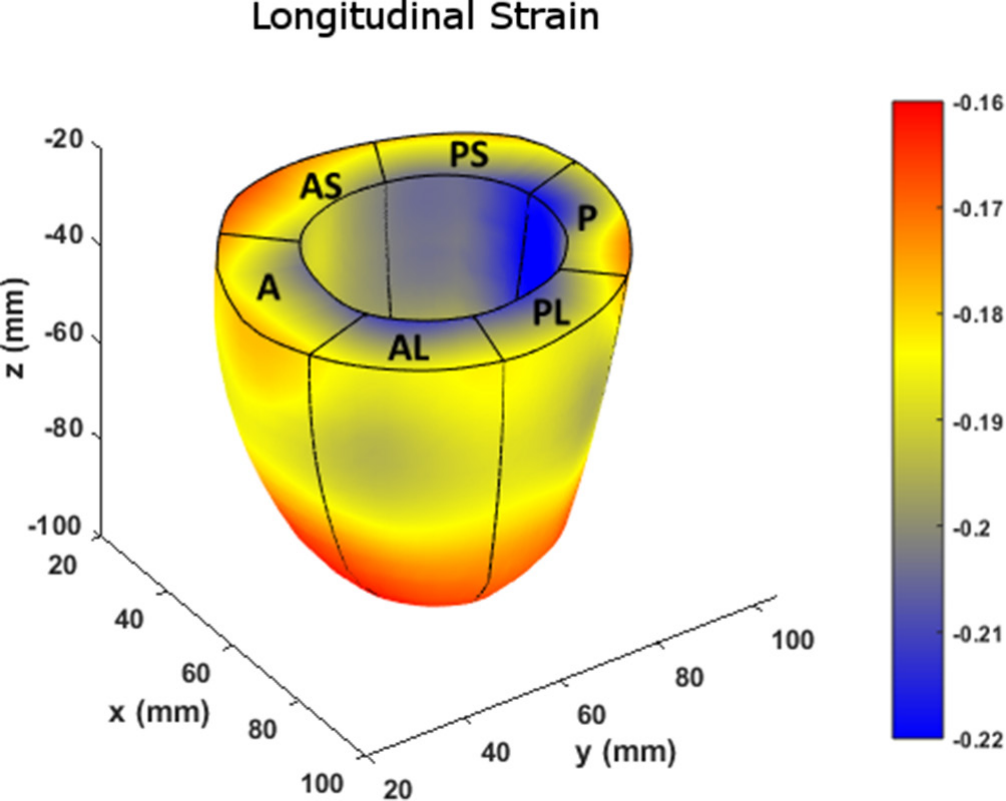
Surface map of longitudinal strain computed with the RPIM method on LV geometries, reconstructed from boundaries detected with the newly introduced 3D semi-automated myocardial contouring technique. Abbreviations: 3D, three-dimensional; A, anterior; AS, anteroseptal; PS, posteroseptal; P, posterior; PL, posterolateral; AL, anterolateral; LV, left-ventricular; RPIM, Radial Point Interpolation Method.

Figure 10 charts the 3D regional strains computed with the two contouring techniques in the 16 standard LV segments. Figures 11–13 shows the 3D surface strain maps in a single subject. Time taken for 3D semi-automated myocardial segmentation was 3.7 ± 1.6 min per subject using the computational capability of a 3.4 GHz Intel Core processor, 16 GB of RAM and a 64-bit operating system. The time taken does not include the time for phase unwrapping and rendering the results of segmentation. Time taken for the Spottiswoode et al segmentation, including phase unwrapping, was typically 10 min and depended on the number of partitions or short-axis slices. The time taken for computing 3D RPIM strains was less than 60 s per subject, irrespective of the method of boundary generation or number of partitions used.

## DISCUSSION

The primary objective of this study was to create an automated mechanism by which to model the 3D LV chamber in its entirety between end-diastole and end-systole, via a mechanism that uniquely tracks the kinematics of 2D slice-based wall motion in each individual (Figures 3–6). To substantiate the feasibility of this new 3D semi-automated methodology for myocardial boundary detection, results of chamber quantifications as well as strain measurements were compared to those generated from a validated 2D method.^9, 10,12^ The results showed the fundamental geometric and functional properties such as LV wall thickness, EDD, ESD, EDV, ESV, EF, LV mass and 3D strains were not significantly different between the measurement types (Table 2 and Figure 10). The similarities in 2D contours that is essential for 3D reconstruction and quantification can be seen in Figures 11–13. Furthermore, the results of chamber quantifications were also compared to those reported in gold-standard steady-state free precision (SSFP) MRI studies.^4, 32,34^ Among the results given in Table 2, diastolic and systolic volumes and ejection fractions are comparable to those given in standard SSFP reports, which are 83–218, 18–82 ml and 57–81%, respectively.^29^ Similarly, LV masses are comparable to SSFP reports with reported values ranging 42–165 g.^29^ Other than chamber quantifications, 3D strain parameters (quantifying material point deformation in the myocardium) were compared and close agreements established between the two contouring techniques (Figures 7–10). In addition, the results of 3D strain analysis are comparable to those reported in existing spatial modulation of magnetization and DENSE-based studies.^12, 15,18,19^ The ability to render surface maps of these 3D strains is shown in Figures 11–13.

Several studies in recent years have emphasized the importance of strain analysis as a subclinical measure for identifying as well as measuring therapeutic progress in cardiac dysfunctions such as ischemia, hypertrophic cardiomyopathy, dilated cardiomyopathy and others.^35–37^ An example of strain-based diagnosis is tracking the reduction in longitudinal strain in the interventricular septal area in hypertrophic cardiomyopathy when EF remains unchanged in the early stages of disease.^38^ This is precisely the type of strain evaluation that can be provided by a single DENSE scan combined with rapid and automated post-processing as demonstrated by this study. Additionally, we have precedence in having used the DENSE sequence for analyzing strain in dilated cardiomyopathy patients where scans were completed with minimum discomfort to patients.^18, 19,39^ In this, the 15 min acquisition window (the timing breakdown for which is given in the acquisition section) can be justified for DENSE as it provides the critical displacement and strain data which a faster SSFP sequence cannot. Furthermore, research is now underway for faster DENSE imaging using technologies such as compressed sense with parallel imaging that deliver high quality images while reducing scan time.^23, 40^

Given next is a summary description of the similarities and differences between the existing 2D method by Spottiswoode et al and the new 3D semi-automated boundary detection method presented here, and also provided in tabulated form in Table 1. At this point, the reader’s attention is drawn to the fact that compared to the 2D Spottiswoode et al boundary detection technique, the semi-automated methodology presented in this study is 3D and does not require LV segment-based initialization for the purposes of boundary generation for every partition. This 3D semi-automated study used the bounding ellipse and radial search to eliminate the need for manually initializing contours for each partition. In contrast, the Spottiswoode et al semi-automated method required short-axis boundaries to be defined for each partition as it essentially lacks boundary initialization.^10, 12^ A second important difference with the 2D Spottiswoode et al method lies in the use of the complementary technique used to refine myocardial extent in addition to phase based displacements. In this context, the Spottiswoode et al method used a modulus deformation mask with which all deformation in excess of the expected myocardial range is rejected, which in turn helped define myocardial extent. Whereas, the new 3D semi-automated technique uses a magnitude image based multithreshold quantization technique and its gradients found in a radial search path to help determine myocardial extent. One of the main similarities to the Spottiswoode et al method lies in regard to finding material point displacements from displacement vectors with tails originating at a reference point on the myocardial boundary and in how only vectors closest in angular orientation and magnitude are used for approximating the location of a new boundary point.^10, 12,18,19,39^ The existing 2D technique also applies some effective noise removing steps associated with motion-based trajectories, including fitting of each trajectory with periodic Fourier series basis functions, which was a technique also used for refining pixelwise trajectories for this study. A final observation made in relation to the comparing the two techniques is the negligible differences in results between them, which can be attributed to conducting this study in healthy young subjects whose ventricular shape and pattern fall within a predefined range. It is hypothesized that there would be higher differences in results between the two techniques in a population with known cardiac disease, in which case significantly more measurement errors could be introduced by a 2D technique that requires more frequent human interactions (at each 2D partition).

The most important limitation of this methodology is the requirement of an ROI (bounding ellipse) at the start of segmentation, where a pattern-based search for a torus to locate the LV in short axis slices will make this algorithm a fully automated one. However, the search for a complex torus shaped LV of non-uniform thickness in an uncropped image can become a computationally demanding process and ultimately prolong the time for generating the 3D contours. Secondly, quantization itself can pose a challenge when the picture quality varies significantly between subsequent timeframes or partitions, in which case, the algorithm must address variations in multithreshold based indices that identify the myocardium, and, in particular, when optimal quantization computations are used for thresholding individual image. A third limitation was the observed difference in fundamental parameters such as LV thickness between 3D semi-automated and Spottiswoode et al contouring techniques in the apical segment (although not indicated by a low *p*-value). The aspects of this limitation are attributed to image degradation induced by cardiac and respiratory motion whose effects include blurring (also ghosting and misregistration) and which in turn effect quantization thresholds.^41^ In this context, if a low resolution blur is quantized below a threshold that identifies intramyocardial tissue, the result will be a reduction in thickness. On the other hand, a blurred tissue–blood interface with high resolution may be erroneously recruited as intramyocardial tissue resulting in a perceived increase in thickness. In order to avoid such anomalies, methods to improve image-based signal degradations need to be addressed with respect to the original sequence and its parameters.^15, 23,41^ Another limitation of this study was in not comparing the current quantization and deformation-based approach to a more traditionally used semi-automation process such as the Active Appearance Model (AAM).^42, 43^ However, there are some apparent drawbacks to AAMs, including the requirement of a training set that can give rise to implausible statistical outliers, and AAMs’ sensitivity to initialization errors can lead to errors related to translation, rotation or scaling.^42–44^ On the other hand, AAM models are built on strong statistical foundations and can withstand substandard quality of data, including missing texture information. In contrast, the current 3D semi-automated technique will fail to model the morphology of the LV if either magnitude or phase information is incomplete. A related limitation is not arduously pursuing other thresholding methods that have been effective for anatomical pattern recognition in medical images. The two commonly used thresholding algorithms, K-mean (each data point is a member of only one cluster) and Fuzzy C-means (a data point can have membership in several clusters) have previously been used in automated detection of the LV lumen but were not considered for this study.^45–47^ A final limitation lies in not conducting gold-standard SSFP studies for LV chamber quantifications. SSFP is the gold-standard measurement technique for chamber quantification and there exists a strong likelihood of seeing significant differences between chamber quantifications by SSFP and the current 3D semi-automated technique.^27, 29,33^ However, SSFP cannot be used for comprehensive LV contraction analysis and is unable provide the displacement data required for the combined purposes of both boundary detection and strain analysis, which is possible with the DENSE-based 3D semi-automated technique.

## CONCLUSION

A novel, semi-automated, quantization and deformation-based technique has been introduced that requires a single ROI to be selected (in a basal diastolic frame) for 3D contouring the full LV. Furthermore, the rapid contouring process achieved within a short time period of less than 3 min is followed by automated chamber quantifications and generation of 3D strain maps. This 3D semi-automated technique was validated by comparison to an existing 2D semi-automated contouring technique and similarities in cardiac chamber quantifications and strain evaluations were seen between the two techniques. A step forward from here will be to validate the current approach in patients with known cardiac dysfunctions such as arrhythmias and cardiomyopathies. A final conclusion is that this new approach can be used to provide rapid ground-truth information regarding 3D cardiac function when data are obtained using a single navigator-gated DENSE scan.

## Declaration

The authors confirm having read the guidance outlined for conflict of interests and indicate that none of the authors have any such conflicts. Additionally, all data related to this project any such conflicts. Additionally, all data related to this project at https:// osf. io/ h5kfd/ DOI: 10.17605/OSF.IO/H5KFD or upon request to the corresponding author, Julia Kar, PhD, email: jkar@ southalabama. edu.
